# A mucin-like peptide from *Fasciola hepatica* instructs dendritic cells with parasite specific Th1-polarizing activity

**DOI:** 10.1038/srep40615

**Published:** 2017-01-12

**Authors:** Verónica Noya, Natalie Brossard, Ernesto Rodríguez, L. Sebastián Dergan-Dylon, Carlos Carmona, Gabriel A. Rabinovich, Teresa Freire

**Affiliations:** 1 Laboratorio de Inmunomodulación y Desarrollo de Vacunas, Departamento de Inmunobiología, Facultad de Medicina, Universidad de La República, Montevideo, Uruguay; 2 Laboratorio de Inmunopatología, Instituto de Biología y Medicina Experimental, Consejo Nacional de Investigaciones Científicas y Técnicas, Buenos Aires, Argentina; 3 Unidad de Biología Parasitaria, Instituto de Higiene, Departamento de Biología Celular y Molecular, Facultad de Ciencias, Universidad de la República, Montevideo, Uruguay; 4 Facultad de Ciencias Exactas y Naturales, Universidad de Buenos Aires, Buenos Aires, Argentina

## Abstract

Fasciolosis is a trematode zoonosis of interest in public health and cattle production. We report here the immunostimulatory effect of a 66 mer mucin-like peptide from *Fasciola hepatica* (Fhmuc), which synergizes with lipopolysaccharide (LPS) to promote dendritic cell (DC) maturation, endowing these cells with Th1-polarizing capacity. Exposure of DCs to Fhmuc in presence of LPS induced enhanced secretion of pro-inflammatory cytokines and expression of co-stimulatory molecules by DCs, promoting their T cell stimulatory capacity and selectively augmenting IFN-γ secretion by allogeneic T cells. Furthermore, exposure of DCs to Fhmuc augmented LPS-induced Toll-like receptor (TLR) 4 expression on the cell surface. Finally, Fhmuc-conditioned DCs induced parasite specific-adaptive immunity with increased levels of IFN-γ secreted by splenocytes from vaccinated animals, and higher parasite-specific IgG antibodies. However, Fhmuc-treated DC conferred modest protection against *F. hepatica* infection highlighting the potent immuno-regulatory capacity of the parasite. In summary, this work highlights the capacity of a mucin-derived peptide from *F. hepatica* to enhance LPS-maturation of DCs and induce parasite-specific immune responses with potential implications in vaccination and therapeutic strategies.

Dendritic cells (DCs) play crucial roles in major functions of the immune response such as central and peripheral tolerance, tissue imprinting and effector immune response towards trauma or infections that might threaten host defenses^1^,^2^. They hold the unique capacity to activate naïve CD4^+^ and CD8^+^ T cells, promoting their differentiation into different effector T cells depending on the nature of the infecting pathogen and the magnitude of infection^2^. This ability relies on their capacity to recognize and identify Pathogen-Associated Molecular Patterns (PAMPs) by specific receptors^3^. Among them, the Toll-like receptor (TLR) family has been the most extensively studied^4^,^5^. Upon encounter of PAMPs with their respective receptors, DCs mature and undergo several processes such as antigen internalization, processing and presentation, along with the activation of signaling cascades directed towards cytokine production^6^. This maturation process results in the increased expression of surface molecules such as MHC class II (MHC II), CD80, CD86 and CD40 which, together endow DCs with improved T cell-stimulatory capacity^6^.

To allow long survival in their hosts, helminth parasites evade host immunity by modifying DC maturation and function^7^,^8^,^9^,^10^, resulting in altered Th2 polarization. Among helminth infections, fasciolosis caused by *Fasciola hepatica*, is of paramount importance due to its wider spectrum of definitive hosts^11^ and its worldwide distribution^12^. Effective chemotherapies are available, but, due to the cost of the treatment and the emergence of resistant strains^13^, alternative methods to control infection such as vaccination, are needed^14^,^15^. To date, vaccination trials with either purified native or recombinant *F. hepatica* proteins have reported a wide range of protection (30–89%) in ruminants^15^,^16^,^17^. Interestingly, it has been proposed that induction of a robust Th1 response could protect the host not only from the infection^15^,^18^ but also from bystander co-infections by down-regulating Th2 regulatory immunity^19^. Accordingly, protection induced by helminth vaccines has been associated with high IFN-γ and TNF production^20^.

Several studies have independently demonstrated that *F. hepatica*-derived molecules inhibit or decrease DC activation inducing a tolerogenic phenotype^21^,^22^,^23^,^24^. Furthermore, DCs from mice infected with *F. hepatica* have a semi-mature phenotype that is characterized by low MHC II and CD40 expression and high secretion of the immunoregulatory cytokine IL-10^25^. Given their remarkable capacity to present antigens to T cells, antigen-loaded DCs have been proposed as vaccines to prevent a spectrum of infectious diseases^26^,^27^,^28^,^29^,^30^. Indeed, DCs pulsed with certain helminths, have been shown to protect against infection, in vaccination regimens^26^. Supporting this concept, bone marrow derived-DCs (BMDCs) loaded with *F. hepatica* antigens in the presence of LPS protected mice against parasite infection^26^.

In this work we focus on the ability of Fhmuc, a mucin-derived synthetic peptide from *F. hepatica*, to interact and modulate LPS-induced DC maturation and to induce protection against *F. hepatica* infection, when administered together with DCs. The 66-mer peptide selected for this study comprises a common sequence of two groups of mucin-like isoforms highly expressed in the newly excisted juveniles (NEJs), the infective stage of *F. hepatica*^31^,^32^. The results presented here show that Fhmuc increases production of LPS-induced pro-inflammatory cytokines and expression of co-stimulatory molecules by DCs. Furthermore, Fhmuc-pulsed BMDCs increased the frequency of parasite-specific IFN-γ-producing splenocytes and production of IgG-specific antibodies in infected animals. Vaccinated animals showed only modest protection, as demonstrated by liver damage, thus emphasizing the immunoregulatory capacity of the parasite. Collectively, this work highlights the immunostimulatory role of a mucin-derived peptide from *F. hepatica*, with potential implications in vaccination strategies against this flatworm.

## Results

### Fhmuc promotes recruitment of CD11c^+^ cells to the peritoneal cavity and favors production of pro-inflammatory cytokines

**Figure 1:**
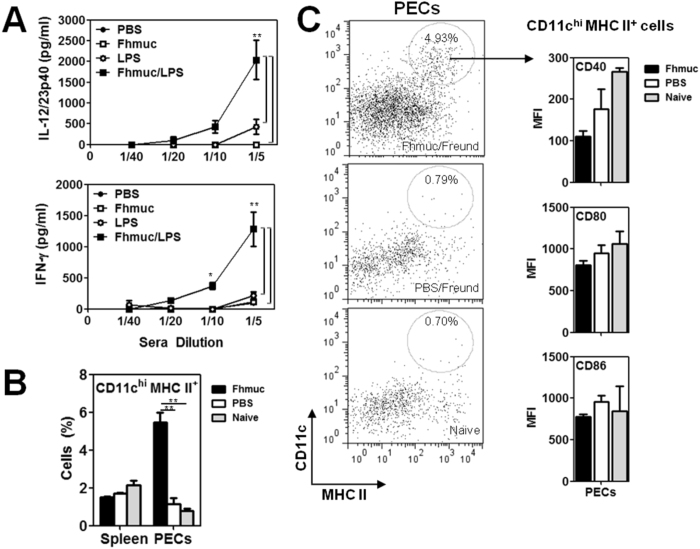
Fhmuc potentiates the production of pro-inflammatory cytokines in a model of septic shock and favors the recruitment of mature CD11c^+^ cells to the peritoneal cavity. (**A**) IFN-γ and IL-12/IL-23p40 production in sera from animals treated with LPS previously injected with Fhmuc or PBS. (**B**) Identification by flow cytometry of CD11c^+^ MHC II^+^ cells in the peritoneum and spleens of animals injected with Fhmuc in Freund’s adjuvant. (**C**) Representative dot plots of CD11c^+^ MHC II^+^ gated cells from PECs of Fhmuc/Freund-, PBS/Freund-injected and naïve mice. The expression of CD40, CD80 and CD86 on CD11c^+^ MHC-II^+^ cells from Fhmuc/Freund-injected animals was evaluated by flow cytometry. MFI stands for Mean fluorescence intensity. Asterisks indicate statistically significant differences (*p < 0.05, **p < 0.001) analyzed by 2 way Anova test.

Given previous data demonstrating the immune-regulatory properties of helminth-derived products (7–8, 10, 15, 18), we first evaluated whether the Fhmuc peptide may down-regulate the production of pro-inflammatory cytokines generated after LPS-induced septic shock. To this end, we injected mice i.p. with PBS or Fhmuc in presence or absence of LPS (0.1 μg) and quantified the concentrations of IL-12/IL-23p40 and IFN-γ in sera after six hours. Sera from LPS-injected animals contained discrete levels of both IL-12/IL-23p40 and IFN-γ (Fig. 1A). Surprisingly, these cytokines were considerably augmented when Fhmuc was injected prior to the administration of LPS, indicating that, in contrast to our original assumption, Fhmuc augmented the production of LPS-induced pro-inflammatory cytokines. These results suggest that Fhmuc peptide exhibited pro-inflammatory properties. Of note, Fhmuc injected in absence of LPS did not induce significant quantities of these cytokines (Fig. 1A), suggesting that Fhmuc may serve to potentiate, but not intrinsically trigger pro-inflammatory responses.

Considering the role of DCs in the initiation of adaptive immunity, we asked whether Fhmuc interacted with these cells or promoted their recruitment to inflammatory sites. Thus, we injected mice with Fhmuc or PBS (control) in the presence of Freund’s adjuvant (as source of TLR agonists) and analyzed CD11c^+^ cells both in the spleen and peritoneal exudates cells (PECs). We detected an increase of CD11c^+^ MHC II^+^ cells in the peritoneal cavity of mice injected with Fhmuc (Fig. 1B and C). Although, these cells expressed similar expression levels of CD40, CD80 and CD86 than control mice (Fig. 1C), a 5-fold increase in CD11c^+^ MHC II^+^ cells was detected in the peritoneal cavity in Fhmuc-inoculated as compared to control mice.

**Figure 2:**
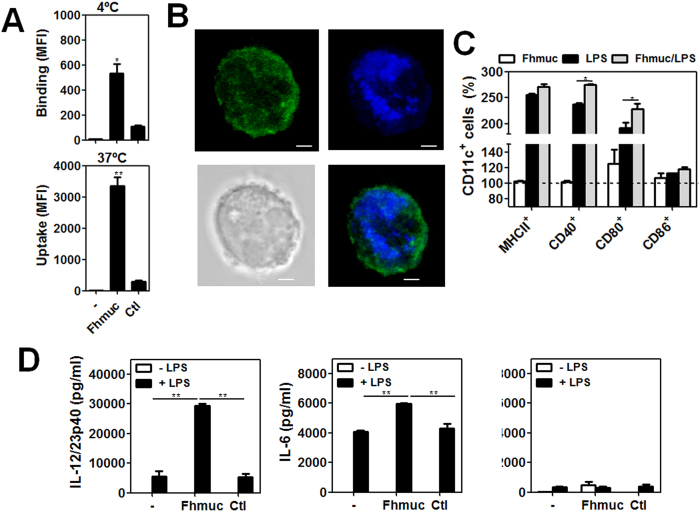
Pro-inflammatory properties of Fhmuc on DCs. (**A**) Fhmuc binding (4 °C) and uptake (37 °C) by DCs as evaluated by flow cytometry on CD11c^+^ gated BMDCs incubated for 1 h with Atto-647-labeled Fhmuc. *p < 0.05, **p < 0.005) analyzed by *t* unpaired test. (**B**) Confocal microscopy of BMDCs incubated for 1 h with Atto-647-labeled Fhmuc (blue) and PE-conjugated anti-mouse CD11c antibody (green). Representative confocal micrographs are shown (scale bar, 1 mm). (**C**) Expression of co-stimulatory molecules and MHC II in BMDCs incubated with Fhmuc (2 h) followed by overnight incubation with or without LPS. Expression of CD11c, MHC II, CD40, CD80 and CD86 was assessed by flow cytometry using specific antibodies. Results are expressed as % in relation with BMDCs incubated in medium alone (100%). (**D**) IL-6, IL-10 and IL-12/IL-23p40 production by BMDCs incubated with Fhmuc (2 h) followed by overnight incubation with or without LPS. As control an irrelevant synthetic peptide was used.

To gain further insights into the possible interaction between Fhmuc and DCs we evaluated the capacity of DCs to bind and/or internalize Fhmuc. We labeled Fhmuc with a fluorescent probe and incubated this fluorescently-labeled peptide with BMDCs either at 4 °C (to assess binding) or at 37 °C (to evaluate uptake). We found that BMDCs bound and internalized Fhmuc as evaluated by flow cytometry (Fig. 2A) and confocal microscopy (Fig. 2B). Given the contribution of Fhmuc to pro-inflammatory responses, we then evaluated whether this mucin-like peptide could modulate DC maturation. When we incubated BMDCs with Fhmuc in the absence of other stimuli, we could see no changes neither in the expression of surface MHC II or co-stimulatory molecules CD40, CD80 or CD86 (Fig. 2C), nor in the secretion of IL-6 or IL-12/IL-23p40 (Fig. 2D). However, when we incubated conditioned BMDCs with Fhmuc followed by the TLR4 agonist LPS, these cells showed higher expression of CD40 and CD80 on their surface (Fig. 2C) and higher secretion of pro-inflammatory cytokines including IL-12/IL-23p40 and IL-6 (Fig. 2D) as compared to BMDCs stimulated with LPS alone. No significant differences were observed in IL-10 production by treated BMDCs.

**Figure 3:**
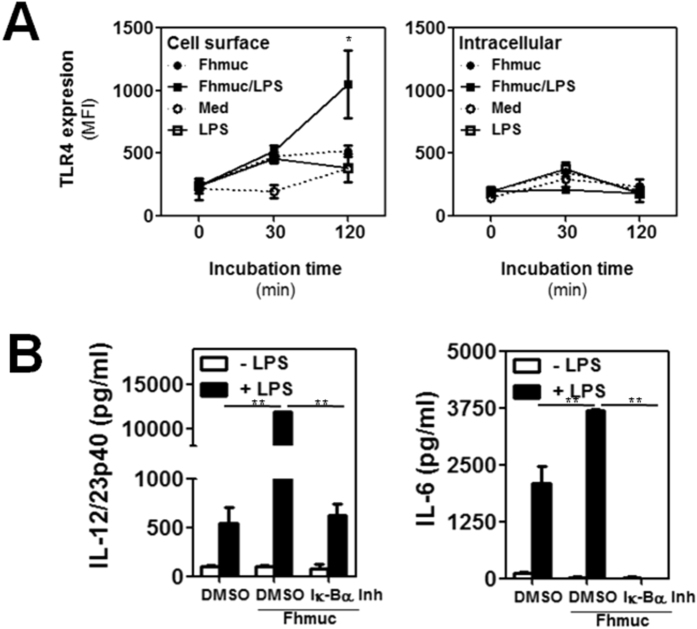
Fhmuc-treated DCs increase TLR4 surface expression and NF-kB signaling. (**A**) Cell surface or intracellular staining of TLR4 in CD11c^+^ BMDCs treated with Fhmuc for 2 h followed by LPS incubation for 0, 30 and 120 min. (**B**) IL-6 and IL-12/IL-23p40 production by BMDCs treated with the IkB-a inhibitor BAY11-7082 followed by treatment with Fhmuc (2 h) and overnight incubation with or without LPS. Results are expressed as the mean values of triplicates (±SD, indicated by error bars) obtained from at least three independent experiments. Asterisks (parts B-F) indicate statistically significant differences (*p < 0.01, **p < 0.001) analyzed by 2 way ANOVA test.

Since Fhmuc enhanced the pro-inflammatory effect of LPS on DCs, but did not induce DC maturation itself, we sought to evaluate whether Fhmuc could induce an increase in the expression of TLR4, the main receptor responsible for mediating LPS recognition and engagement of pro-inflammatory signaling pathways leading to activation of the NF-κB transcription factor^4^,^33^,^34^. Thus, we treated BMDCs with Fhmuc followed by LPS, or with Fhmuc or LPS alone and evaluated the kinetics of TLR4 expression by flow cytometry. When BMDCs were pre-conditioned with Fhmuc followed by LPS we did not observe major changes in the intracellular expression of TLR4 (Fig. 3A). However, when DCs were incubated with Fhmuc we observed an increase in surface expression of TLR4 at 30 min post-stimulation. Furthermore, after conditioning DCs with Fhmuc/LPS, we observed a considerable increase in surface expression of TLR4 compared to BMDCs incubated with LPS alone (Fig. 3A). To explore the involvement of NF-κB in signaling induced by Fhmuc/LPS on DCs, we incubated DCs with the above mentioned stimuli in the absence or presence of BAY11-7082, a specific IκB-α inhibitor. As shown in Fig. 3B, IκB-α inhibition abrogated the production of IL-12/IL-23p40 and IL-6 induced by Fhmuc/LPS on DCs.

### DCs conditioned with Fhmuc and LPS show increased T-cell stimulatory capacity and helminth-specific Th1 promoting activity

**Figure 4:**
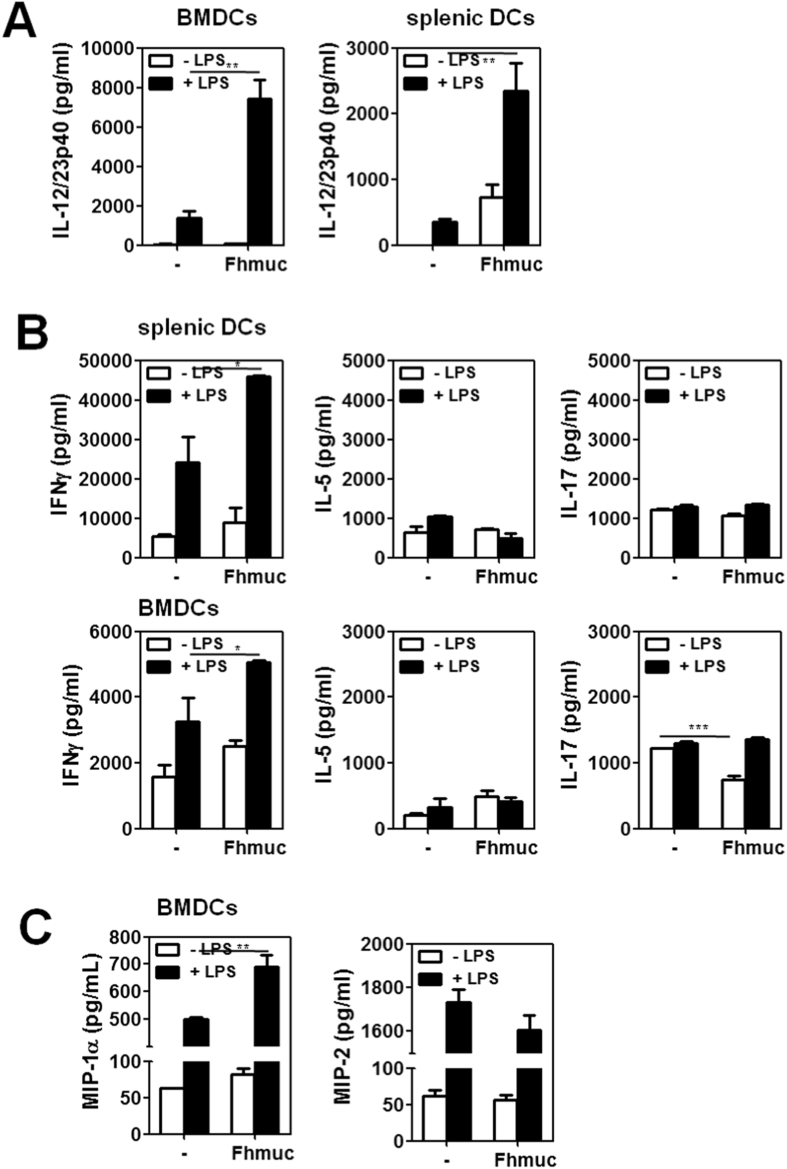
Allostimulatory capacity of Fhmuc/LPS-conditioned DCs. (**A**) IL-12/IL-23p40 production by Fhmuc/LPS-stimulated BMDCs or purified splenic DCs. (**B**) IFN-γ, IL-5 and IL-17 production by BALB/c splenocytes incubated for 72 h with Fhmuc/LPS-treated C57BL/6 splenic DCs or BMDCs in a 5:1 relation. (**C**) MIP-1α and MIP-2 chemokine production by Fhmuc/LPS-stimulated BMDCs. Results are expressed as the mean values of triplicates (±SD; indicated by error bars) obtained from at least two independent experiments. Asterisks indicate statistically significant differences (*p < 0.05, **p < 0.01, ***p < 0.001) analyzed by 2 way ANOVA test.

We then evaluated whether Fhmuc-treated DCs could stimulate T cells in a mixed lymphocyte reaction. To this end, we evaluated LPS-induced DC maturation in the presence of Fhmuc both in BMDCs and purified splenic DCs. When stimulated with Fhmuc in presence of LPS these cells secreted higher levels of IL-12/IL-23p40 as compared to LPS-treated DCs (Fig. 4A). In addition, co-culture of Fhmuc/LPS-treated BMDCs or splenic DCs with allogeneic splenocytes resulted in production of significantly higher levels of IFN-γ as compared to DCs stimulated with LPS alone (Fig. 4B), suggesting a Th1-like polarizing ability of Fhmuc/LPS-conditioned DCs. Of note, no changes in the production of IL-5 were observed, while IL-17 production was decreased when DCs were stimulated with Fhmuc alone (Fig. 4B).

The pro-inflammatory chemokines MIP-1α and MIP-2 are produced by DCs and contribute to regulate the influx of inflammatory cells by selectively attracting Th1 and Th2 cells, respectively [ref. 27 and 28]. We found that DCs stimulated with LPS produced both chemokines. When incubated with Fhmuc/LPS the production of MIP-1α significantly increased (Fig. 4C), thus substantiating a specific role for Fhmuc in favoring LPS-induced Th1-polarization.

**Figure 5:**
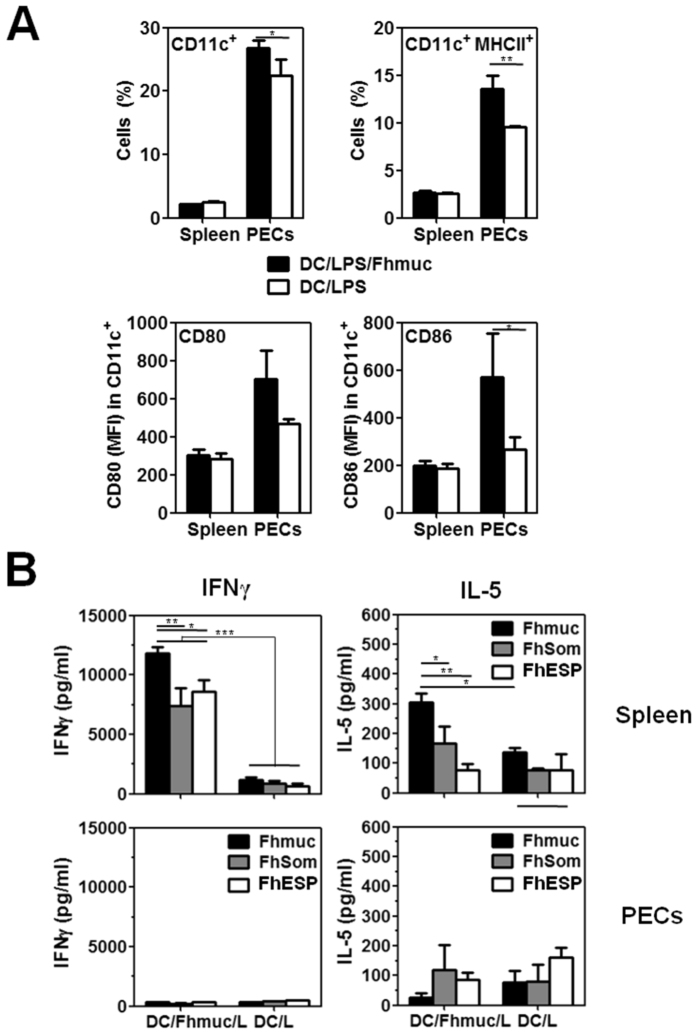
T-cell responses induced *in vivo* by Fhmuc/LPS-stimulated DCs. (**A**) Identification of CD11c^+^ MHC II^+^ cells in the spleen and peritoneal cavity of animals injected with Fhmuc/LPS-stimulated BMDCs (1 × 10^6^ cell/mouse). CD80 and CD86 expression (MFI) on CD11c^+^ gated cells was also evaluated. (**B**) *In vivo* T-cell response triggered by Fhmuc/LPS-stimulated DCs. IFN-g and IL-5 production was evaluated by ELISA in cell cultures of splenocytes or PECs from DC/Fhmuc/LPS-inoculated mice re-stimulated with Fhmuc (10 μg/ml), FhESP (10 μg/ml), or FhSom (100 μg/ml). Results are expressed as the mean values of triplicates (±SD, indicated by error bars) obtained from at least three independent experiments. Asterisks indicate statistically significant differences (*p < 0.05, **p < 0.01, ***p < 0.001) analyzed by 2 way ANOVA test.

Next, we evaluated the stimulatory capacity of Fhmuc/LPS-stimulated DCs *in vivo*. To this end, we injected i.p. Fhmuc/LPS-conditioned BMDCs and analyzed their phenotype both in the spleen and in the peritoneal cavity. As shown in Fig. 5A, CD11c^+^ cells from the peritoneum of mice injected with Fhmuc/LPS-treated BMDCs expressed higher levels of MHC II and the co-stimulatory molecule CD86. Moreover, Fhmuc/LPS treated BMDCs were capable of stimulating the secretion of IFN-γ by Fhmuc-stimulated splenocytes (Fig. 5B). To evaluate whether T cells primed *in vivo* with Fhmuc/LPS treated-BMDCs could recognize parasite antigens, we stimulated splenocytes from injected animals with a protein lysate containing total *F. hepatica* antigens (FhSom) or with excretion-secretion parasite products (FhESP). When stimulated with parasite antigens, splenocytes primed *in vivo* with Fhmuc/LPS-BMDCs produced high levels of IFN-γ and very low amounts of IL-5, indicating that BMDCs stimulated with Fhmuc followed by LPS can prime splenocytes *in vivo* toward a Th1 profile capable of reacting with parasite antigens.

### Fhmuc/LPS-treated DCs induce increased parasite-specific IgG antibodies and IFN-γ-secreting splenocytes in vaccinated animals

**Figure 6:**
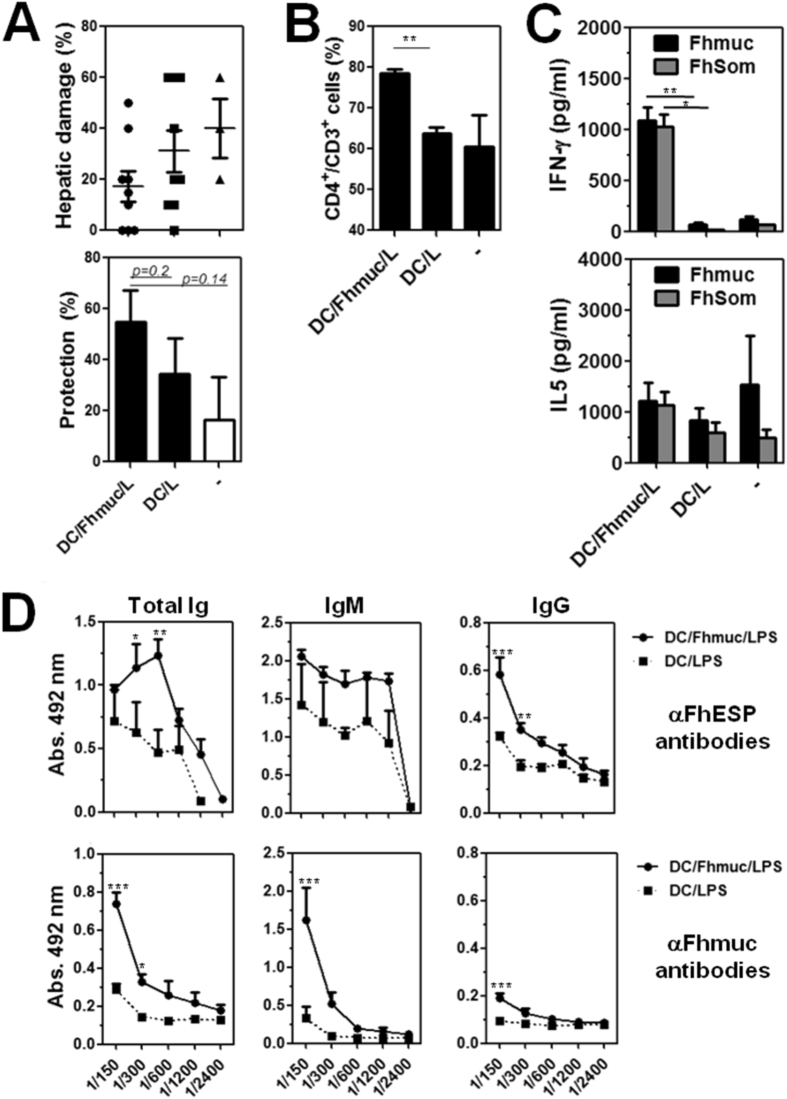
Vaccination with DC conditioned with Fhmuc and LPS leads to modest reduction of liver damage, increased parasite-specific Th1 response and augmented antibody titers. (**A**) Protection from liver damage in DC/Fhmuc/LPS- and DC/LPS-vaccinated as well as control mice. Liver damage was evaluated in liver tissue sections stained with haematoxylin/eosin considered as the percentage of tissue area with lymphocyte infiltration, necrosis or hydropic degeneration. Protection was calculated according to the liver damage attributed to DC/Fhmuc/LPS- and DC/LPS-vaccinated mice, in relation to damage of infected animals that did not receive any treatment (control). (**B**) CD4^+^ CD3^+^ T cells (%) in the spleens of DC/Fhmuc/LPS- and DC/LPS-vaccinated mice and control animals as evaluated by flow cytometry. (**C**) IFN-γ and IL-5 production by splenocytes from DC/Fhmuc/LPS- and DC/LPS-vaccinated as well as control mice. Cells were incubated with Fhmuc (10 μg/mL) or FhSom (100 μg/ml) for three days. Cytokines were quantified by ELISA. (**D**) Reactivity of sera from DC/Fhmuc/LPS- and DC/LPS-vaccinated mice. Antibody reactivity was evaluated on plates coated with Fhmuc or FhESP (1 μg/well). Results are expressed as the mean values of triplicates (±SD, indicated by error bars) obtained from at least two independent experiments. Asterisks (parts B-F) indicate statistically significant differences (*p < 0.05, **p < 0.01, ***p < 0.001) analyzed by 2 way ANOVA test.

Finally, we evaluated whether administration i.p. of Fhmuc-treated DCs could prevent or reduce liver damage following *F. hepatica* infection. Mice vaccinated with Fhmuc/LPS-treated BMDCs showed reduced liver damage and higher degree of protection as compared to control mice (vaccinated with PBS/LPS-treated BMDCs), although these differences were not statically significant (Fig. 6A). Interestingly, partial protection was associated with a higher percentage of CD3^+^CD4^+^ T cells in the spleen (Fig. 6B) and with higher capacity of splenocytes to produce IFN-γ when stimulated with either Fhmuc or parasite molecules (Fig. 6C). Furthermore, DC/Fhmuc/LPS-vaccinated mice presented higher titers of anti-*F. hepatica* IgM and IgG antibodies than control animals (Fig. 6D).

## Discussion

In this work we show that a mucin-derived peptide from *F. hepatica* can enhance LPS-triggered maturation of DCs, and can stimulate *F. hepatica*-specific T cell responses when associated to a Th1-driven stimulus such as LPS. However, these immune responses were not sufficient to mediate total protection from liver damage in vaccinated-mice. The fact that this peptide is highly expressed in the NEJ infective stage of the parasite, suggests that it could be a good vaccine candidate, since a vaccine targeting the NEJ, able to reduce invasion of the liver parenchyma, would minimize liver damage and pathology. In addition to its pro-inflammatory properties in *in vitro* cultures, when administrated i.p., Fhmuc favored the recruitment of CD11c^+^ MHC II^+^ cells to the peritoneal cavity. The chemotactic characteristics of mucins have already been reported. In fact, tumor-derived mucins attract immature DCs *in vitro*^35^. In order to exert their functions, chemoattractants must bind to a specific receptor on the cell surface, which in turn confers cells the ability to detect and move toward a chemotactic stimulus. In this regard, we were able to determine binding of Fhmuc to the DC surface. Human or parasite mucins have been shown to bind to DCs through different C-type lectin receptors via specific glycan structures^9^,^35^,^36^,^37^,^38^. However, the Fhmuc peptide used in this work was unglycosylated, thus precluding the involvement of lectin-glycan interactions in this process.

Fhmuc also enhanced the stimulatory capacity of DCs and their ability to polarize T cell responses toward Th1 profiles likely by increasing the production of LPS-induced IL-12/IL-23p40, IL-6 and MIP-1α, and augmenting the expression of TLR4. The fact that the observed increase in the expression of TLR4 was confined to the cell surface and not to the intracellular compartment, suggests that Fhmuc could stabilize the TLR4 complex at the DC surface. Thus, in the presence of Fhmuc, more potent interactions between DCs and LPS could operate, which could elicit stronger TLR4 signaling leading to the production of pro-inflammatory molecules through NF-κB activation. Interestingly, Fhmuc in absence of LPS induced the secretion of IL-12/IL-23p40 by splenic DCs but not by BMDCs, suggesting that splenic DCs have a higher capacity to polarize T cells toward a Th1 profile.

Fhmuc-conditioned DCs were also capable of priming parasite-specific T cells *in vivo*, indicating not only the potent stimulatory capacity of DCs, but also the specificity of Fhmuc for parasite products. This fact was also associated with higher levels of CD11c^+^ MHC II^+^ cells in the peritoneum of injected animals, suggesting recruitment of these cells to sites of injection. When Fhmuc-conditioned DCs were used to vaccinate mice, they were capable of inducing IFN-γ-producing splenocytes and higher titers of specific IgG antibodies. Nevertheless, the protection in terms of liver damage was modest, indicating that Th1 immunity induced by Fhmuc/LPS-treated BMDCs was not sufficient to induce full parasite protection. We have recently reported that *F. hepatica* infected mice vaccinated with the Fhmuc peptide in Freund’s adjuvant exhibited reduced liver damage than control mice^39^ and that protection was associated with the recruitment of B and T lymphocytes and eosinophils to the peritoneum, independently of mature DCs^39^. In the light of these results we suggest that eosinophils and B or T cells might have a role in the Fhmuc-induced protection since mice inoculated with Fhmuc/LPS-treated BMDCs did not show recruitment of these cells (not shown). Finally, and also considering that DC-loaded with molecules from *F. hepatica* were previously able to protect infected mice from liver damage^26^, we might speculate that Th1 immunity against multiple parasite antigens is more effective to obtain higher degrees of protection.

It is worth noting that different *F. hepatica* antigen preparations were not capable, by themselves of eliciting DC signaling and promote DC maturation. In contrast, many of them inhibited TLR-induced DC maturation^21^,^22^,^23^,^24^,^25^,^40^. However, as reported by Falcon and colleagues^26^, the inhibitory effect exerted by *F. hepatica* molecules could be reverted when treatment with LPS was delayed. In spite of this fact, other *F. hepatica* protein preparations, such as those from the tegument, were not capable of augmenting IL-12 production^24^, suggesting that modulation of DC maturation depends on the time of exposure to a given stimulus, as well as on the composition of the antigens added to cell cultures. Our results suggest that Fhmuc, as FhSom, augments the capacity of LPS to induce DC maturation. Notably, studies on DC immunomodulation were conducted using *F. hepatica* components from the adult stage of the parasite, whereas Fhmuc is particularly abundant in juveniles^32^,^31^.

In conclusion, DC pulsed *ex vivo* with Fhmuc followed by LPS stimulation promote a maturation phenotype and endow DCs with the capacity to prime a systemic Th1 response and enhance specific antibody responses in vaccinated mice. Although these effects might contribute to parasite eradication in the peritoneal cavity or during early stages of migration through the liver, further studies are needed to evaluate the protective capacity of Fhmuc-treated DCs in combination with other parasite antigens. From a broader perspective, Fhmuc may be included in vaccinations protocols to amplify Th1 protective responses triggered by conventional adjuvants.

## Methods

### Ethics statement

Mouse experiments were carried out in accordance with strict guidelines from the National Committee on Animal Research (Comisión Nacional de Experimentación Animal, CNEA, http://www.cnea.org.uy, National Law 18.611, Uruguay) according to the international statements on animal use in biomedical research from the Pan American Health Organization (PAHO) and World Health Organization (WHO). Adult worms were collected during the routine work of a local abattoir (Frigorífico Carrasco) in Montevideo (Uruguay). Protocols were approved by the Uruguayan Committee on Animal Research (Comisión Honoraria de Experimentación Animal, CHEA Protocol Numbers: 071140-000443-10 and 071140-000143-12).

### Mice

Six- to 8-week-old female C57BL/6 mice were obtained from DILAVE (Uruguay) and housed with water and food supplied *ad libitum* under standard lighting conditions (12/12 hours ligh/dark). Animals were handled in accordance with institutional guidelines for animal welfare by the Committee on Animal Research (CHEA, Uruguay).

### Fhmuc peptide

A 66 amino acid peptide of a *F. hepatica* mucin like-protein (corresponding to a part of predicted protein of contig FH00023), named Fhmuc^39^, was chemically synthesized by Peptide 2.0 Inc. (Virginia). The amino acid sequence of this peptide, Fhmuc, is H_2_N-VSSDASTTSTTMTARSSSASATASSETRAPSSTMTTQNASTTSGSVRLPIQTTRCL LFIFGVAFF-COOH. Very low levels of endotoxins were determined in Fhmuc using the Limulus Amebocyte Lysate kit Pyrochrome (Associates of Cape Cod, Massachusetts) (<0.003 EU/μg protein) in agreement with the lack of DC maturation induced by Fhmuc alone.

### Septic shock

Mice were injected i.p. with either Fhmuc (10 μg) or phosphate saline buffer (PBS). After two hours septic shock was induced by administrating i.p. LPS from Escherichia *coli* 0111:B4 (Sigma-Aldrich, Missiouri) at 1 μg/mouse. Six hours later animals were bled and euthanized. Pro-inflammatory cytokines such as IL-12/IL-23p40 and IFN-γ were evaluated in sera by ELISA (BD Biosciences, California).

### Preparation of excretion/secretion products (FhESP) and total lysate (FhSom)

Live adult worms were obtained from the bile ducts of bovine livers and then washed for 1 h at 37 °C with PBS (pH 7.4) and used to prepare FhESP and FhSom as previously described^39^. Endotoxins were removed using Detoxi-Gel Endotoxin Removing Gel (Thermo Fisher Scientific Inc., Illinois). The protein concentration of parasitic lysates was measured using a bicinchoninic acid assay (Sigma-Aldrich, Missiouri). Endotoxin levels were determined using the Pyrochrome Limulus Amebocyte Lysate kit (Cape Cod Inc., Massachusetts).

### Immunization with Fhmuc

Mice (5/group) were immunized intraperitoneally (i.p.) with Fhmuc (20 μg) or PBS (control group) in complete Freund’s adjuvant on day 0 followed by two additional injections in incomplete Freund’s adjuvant on days 14, and 28. Three independent experiments were carried out. Mice were sacrificed by cervical dislocation on days 42 and spleens and peritoneal exudates cells (PECs) were removed^39^. Cells were stained with antibodies to identify DCs including anti-CD11c (N418), -CD40 (HM40-3), -MHC II (m5/114.15.2), I-A/I-E (2G9), -CD80 (16-10A1), and -CD86 (GL1) antibodies, and analyzed using a CyAn ADP Analyzer (Beckman Coulter, California). Antibodies were obtained from eBioscience or from BD Biosciences (California).

### Generation of BMDCs

BMDCs were generated from C57BL/6 bone marrow precursors (2 × 10^5 ^cells/ml) that were plated in complete culture medium (RPMI-1640 with glutamine supplemented with 10% FBS, 50 μM 2-mercaptoethanol, 100 U/ml penicillin and 100 mg/ml streptomycin) supplemented with a GM-CSF-containing supernatant. Cells were recovered on day 7 and analyzed by flow cytometry using antibodies specific for CD11c, CD40, CD80, CD86, MHC-II, CD11b (M1/70), F4/80 (BM8). Cytometry analyses revealed a purity of at least 95% of CD11c^+^ cells.

### Purification of splenic DCs

Splenic DCs were purified using the Miltenyi-Biotec MACS system (MicroBeads mouse) from spleens collected from naïve mice, according to the manufacturer’s recommended protocol. Cytometry analyses revelead a purity of at least 90% of CD11c^+^ cells.

### Maturation of DCs

BMDCs or splenic DCs (25 × 10^4^ cells/well) were pulsed with serial dilutions of Fhmuc (0–10 μg/ml), for 2 h and further incubated with LPS (1 μg/ml) overnight at 37 °C. The culture supernatants were frozen, and then tested for IL-6, IL-10, IL-12/IL-23p40, MIP-1α and MIP-2, measured by specific ELISA assays by BD Bioscience or RayBiotech Inc. (Gerogia, USA). Alternatively, BMDCs were incubated with 10 mM of the Iκ-Bα inhibitor Bay11-7082 (Invivogen, California) diluted in DMSO or DMSO (control) for 1 h prior to the Fhmuc/LPS incubation. Cell viability was assessed with Trypan blue exclusion test. Controls included BMDCs incubated with an irrelevant peptide corresponding to a sequence of a human mucin MUC6 (PLITVTTSRTSQVHS) or an *Echinococcus granulosus* mucin (MSLLSPSTPLHAITS) at 10 μg/ml.

### Flow cytometry for cell surface phenotype

Splenocytes or PECs from DC/Fhmuc/LPS or DC/LPS-vaccinated mice were washed twice with FACS buffer and stained with antibodies to identify B and T cells [anti-CD3 (17A2), -CD4 (RM4-5), -CD8α (53-6.7), and -CD19 (eBio1 D3)] and DCs or macrophages (anti-CD11b, -CD11c, -CD40, -MHC-II, -F4/80, -CD80, and -CD86) by flow cytometry. Antibodies were obtained from eBioscience or BD Biosciences.

### Flow cytometry for TLR4 expression

BMDCs (1.0 × 10^6^ cell/well) were distributed in 96-well culture plates in the presence or absence of Fhmuc (10 μg/ml) for 2 hrs at 37 °C 5% CO_2_. Then, LPS (1.0 μg/ml) was added and incubated for 30, 60 and 120 min. Cells were then stained with anti-CD11c, permeabilized and incubated with PE-anti-mouse CD284 (TLR4, Biolegend, California) prior to flow cytometry analysis.

### Mixed lymphocyte reaction (MLR)

Fhmuc-conditioned DCs (C57BL/6, 0.2 × 10^6^ cells/well) were washed three times in PBS and incubated with splenocytes (1 × 10^6^ cells/well) from BALB/c mice. Cell proliferation was evaluated according to [^3^H]-thymidine incoporation (CPM). Culture supernatants were collected and analyzed by ELISA for IFN-γ, IL-5 and IL-17 production.

### *In vitro* Fhmuc binding and internalization assay

BMDCs (2.5 × 10^5^/well) were incubated with Alexa 647-labeled-Fhmuc for 1 h at 37 °C in complete medium (to assess uptake), or at 4 °C in complete medium (to assess binding). Cells were then stained with anti-CD11c antibody, washed twice with FACS buffer and analyzed by flow cytometry. Controls included BMDCs incubated with an irrelevant peptide corresponding to a sequence of a human mucin MUC6 (PLITVTTSRTSQVHS) or an *Echinococcus granulosus* mucin (MSLLSPSTPLHAITS) at 10 μg/ml. Internalization was also analyzed by confocal microscopy on stained cells that were fixed with 1% formaldehyde and dried over glass plates. Cells were then stained with PE-conjugated anti-mouse CD11c antibody. Slides were visualized in a Spectral Confocal Microscope Leica TCS SP5.

### T cell response in mice inoculated with DC/Fhmuc/LPS

Mice (8/group) were inoculated i.p. with Fhmuc-loaded BMDCs followed by LPS stimulation (DC/Fhmuc/LPS, 1 × 10^6^ cells/mouse). Control mice received LPS-stimulated BMDCs (DC/LPS). Two weeks later, mice were bled, sacrificed and spleens or PECs were removed, washed and suspended (1 × 10^6^ cells/well) in complete culture medium. Cells were incubated in 96-well plates with Fhmuc (10 μg/ml), FhSom (100 μg/ml), FhESP (10 μg/ml) for 72 h at 37 °C with 5% CO_2_. Secreted cytokines (IFN-γ, IL-5, and IL-17) were measured in culture supernatants by sandwich ELISAs (BD Biosciences).

### Vaccination of mice with DC/Fhmuc/LPS

For vaccination assays, two groups of at least 8 mice per group were used. Animals were vaccinated i.p. with DC/Fhmuc/LPS (1 × 10^6^ cells/mouse). Control mice received LPS-stimulated BMDCs (DC/LPS). Two weeks later, mice were infected with 10 *F. hepatica* metacercariae/mouse (Baldwin Aquatics, USA). Following three weeks of infection, mice were bled, sacrificed by cervical dislocation, and the livers, spleens and PECs were removed. Livers were embedded in paraffin to perform histological analysis. Paraffin sections were cut from livers and stained with haematoxylin-eosin. Histological analysis of liver sections was evaluated by a trained pathologist in a blind way. Liver damage in multiple sections of hepatic tissues representative of the organ was determined according to the percentage (%) of affected area. The affected area was taken into consideration if lymphocyte infiltration, hydropic degeneration or necrosis were detected^39^. Liver sections from uninfected and non-vaccinated infected animals served as control groups. The percentage (%) of the affected area was used to calculate the protection that corresponded to 100% in absence of liver damage, or to 0% when there was more than 60% of damaged liver tissue, as previously described^39^.

Three independent vaccination experiments were carried out.

### Evaluation of immune response in DC/Fhmuc/LPS-vaccinated animals

Splenocytes or PECs (2.5–5 × 10^6^ cells/ml) from DC/Fhmuc/LPS-vaccinated animals were cultured in complete medium for 72 h in the presence or absence of Fhmuc peptide (10 μg/ml), FhSom (100 μg/ml) or FhESP (10 μg/ml). Secreted cytokines (IFN-γ, IL-5, and IL-17) were measured in culture supernatants by sandwich ELISAs (BD Biosciences). Sera reactivity against Fhmuc or FhESP was analyzed by ELISA as described^39^. Negative control consisted of sera from mice injected with PBS in adjuvant diluted 100-fold.

### Statistical Analysis

Unpaired parametric *t* and 2-way Anova tests performed on GraphPad Prism version 5.01 were used for statistical comparisons. *P* values of <0.05, <0.01 or <0.001 were considered to be statistically significant, depending on the experiment.

## Additional Information

**How to cite this article**: Noya, V. *et al*. A mucin-like peptide from *Fasciola hepatica* instructs dendritic cells with parasite specific Th1-polarizing activity. *Sci. Rep.* **7**, 40615; doi: 10.1038/srep40615 (2017).

**Publisher's note:** Springer Nature remains neutral with regard to jurisdictional claims in published maps and institutional affiliations.
